# Evolution and Development of Dual Ingestion Systems in Mammals: Notes on a New Thesis and Its Clinical Implications

**DOI:** 10.1155/2012/730673

**Published:** 2012-09-18

**Authors:** Jeffrey R. Alberts, Rita H. Pickler

**Affiliations:** ^1^Department of Psychological and Brain Sciences, Indiana University, Bloomington, IN 47405, USA; ^2^Cincinnati Children's Hospital Medical Center, 3333 Burnet Avenue, MLC 11016, Cincinnati, OH 45229, USA

## Abstract

Traditionally, the development of oral feeding is viewed as a continuous, unitary process in which reflex-dominated sucking behavior gives rise to a more varied and volitional feeding behavior. In contrast, we consider the thesis that the infant develops two separable ingestive systems, one for suckling and one for feeding. First, we apply an evolutionary perspective, recognizing that suckling-feeding is a universal, mammalian developmental sequence. We find that in mammalian evolution, feeding systems in offspring were established prior to the evolution of lactation, and therefore suckling is a separable feature that was added to feeding. We next review an experimental literature that characterizes suckling and feeding as separable in terms of their topography, sensory controls, physiological controls, neural substrates, and experience-based development. Together, these considerations constitute a view of “dual ingestive systems.” The thesis, then, is that suckling is not a simple precursor of feeding but is a complete behavior that emerges, forms, and then undergoes a dissolution that overlaps with the emergence of independent feeding. This thesis guides us to focus differently on the challenges of properly managing and facilitating oral ingestion in infants, especially those born preterm, prior to the developmental onset of suckling.

## 1. Introduction

The development of oral ingestion in mammalian infants proceeds in a distinct and invariant sequence: suckling from a nipple is followed by a transition to independent feeding. Conventional wisdom is that early ingestion (suckling) is dominated by reflexes and by centrally generated behavior patterns that give rise to a more complex, varied, and volitional form of ingestion (feeding). Thus, the development of oral ingestion is viewed as unitary, developmental process. This paper offers a different view of the development of oral ingestion. Put simply, our thesis is that in every mammal there are at least two, separable ingestion systems, namely, the suckling system and the feeding system. Each component of this dual ingestion system can be differentiated by its evolutionary origin, because *mammalian ingestion evolved twice*. Similarly, each component of this dual ingestion system can be differentiated developmentally because *oral feeding develops twice in mammals*. As such, proper and appropriate developmental care is aimed at protecting, supporting, and facilitating the development of two separable and specialized feeding systems—the development of suckling and the development of independent feeding.

To the parents, nurses, therapists, and physicians who care for prematurely born infants, this developmental sequence is particularly prominent, vital, and often fraught with difficulties. Indeed, infants younger than 32 weeks postconceptional age are often incapable of orally grasping a nipple and sucking, to say nothing of coordinating sucking with breathing and swallowing [1–4]. Caregivers of infants born preterm then provide life-sustaining support while anticipating the best time to initiate oral ingestion (suckling) and then applying a variety of techniques to facilitate the further development of ingestion [5–7] so that the infant can be discharged home where continued development of feeding behavior will occur.

In the present paper, we follow the terminological rules suggested by Hall et al. [8] for discussing the processes involved in milk transfer from mother to offspring. *Lactation* refers to the physiological state associated with milk production and secretion. *Nursing* comprises the behavioral and physiological activities that promote milk transfer to offspring. *Suckling* refers to the behavior of the young that contribute to the receipt of milk from a nipple or teat. *Sucking* consists of the oral motor movements that typically produce the intraoral pressures that help express milk from the nipple (see [8, 9] for a more complete discussion and definitions). In addition, *feeding* refers to the oral ingestion of nutritive substances other than milk, usually but not necessarily in solid or near-solid forms that typically are chewed. Finally, *ingestion* is used to encompass all forms of oral intake and includes both suckling and feeding.

We will briefly describe the *evolution* of lactation and mammalian ingestion and its relationship to suckling and feeding in the human. We will then summarize a set of *developmental* analyses from nonhuman research that compare a variety of perspectives on suckling and feeding behavior. Finally, we will identify some implications for a research agenda and for improving outcomes in children who are born preterm.

## 2. The Evolutionary Context of Lactation and Suckling Young

The vocabulary of geological timescales will be used to frame and discuss the evolution of mammals, the origins of their lactation, and the evolution of suckling offspring. We will take a rapid and abbreviated trip through geological time, sampling representative evidence that constitutes some of the fundamental understanding of mammalian origins. Within the timeline of the story, we will note the evidence pertinent to the evolution of lactation. This is uncertain territory, but we can draw liberally from a remarkable synthesis of paleontological, physiological, histological, and comparative data, recently proffered by Oftedal [10, 11] and others that provides a coherent and exciting picture of when and how lactation may have emerged to become a unique, universal, and defining feature of mammalian life.

Table 1 shows some standard divisions [12, 13] arranged chronologically from the oldest (bottom) to most recent (top). At the base of Table 1 is the Cambrian period, over 500 million years ago (mya), the period yielding the oldest known fossils of vertebrates [14]. Cambrian vertebrates were aquatic, eel-like creatures, reflected today in the jawless hagfish. These early forms proliferated and differentiated, giving rise to new types of aquatic life, such as the armoured fishes of the Silurian and Devonian periods. Reproductively, Cambrian vertebrates produced eggs that were fertilized externally. Parental care was unknown, so the offspring emerged from the egg case ready to live and feed independently. By the Carboniferous period (360 mya), there was a much greater diversity of vertebrate types. With diverse morphology came diversity in habitats and habits. Vertebrate life found its way from the aquatic medium into the air, some became amphibious, and others adopted fully terrestrial habits. Locomotion and diet evolved along with many changes in skeletal structures, appendages, and skull structure.

### 2.1. The Amniote Egg and the Path toward Mammals

During the Pennsylvanian, around 300 mya, there appeared a developmental innovation that proved to be a pivotal point in vertebrate evolution—the amniote egg, distinguished by additional extraembryonic membranes as well as outer layers that improved gas exchange, utilization of nutrients, and water retention [15]. Such features made it possible for the amniote-producing tetrapods to move into previously untapped habitats and accomplish successful reproduction and development. The Pennsylvanian fossil record contains a number of different amniotes that branched into separate lines, which differ by the presence and number of skull windows (fenestrae). Those lacking a skull window, or with two fenestrae, comprise the Sauropsida. One of these amniotes led to the branches comprising the turtles and another to the Diapsids that would diversify into today's squamate species, which include all lizards and snakes. The amniotes with one fenestra constitute the Synapsida. This is the line that would become the predecessor of all the mammals on Earth. Estimates are that these amniotic eggs began appearing about 310 mya.

**Figure 1:**
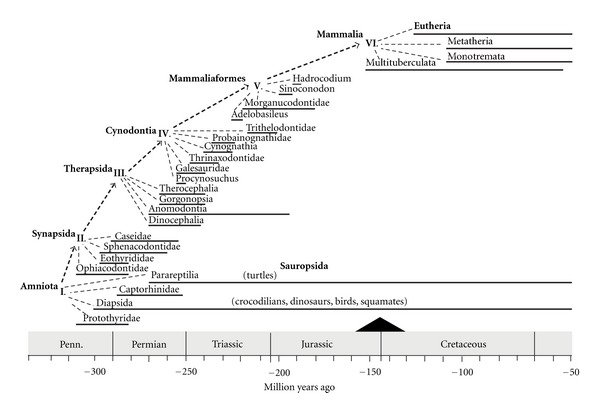
A simplified representation of some of the sequential, evolutionary radiations that appear from Amniota (I.) to Mammalia (II.) showing, for comparative purposes, the cladistic separation of the synapsids and saurosids as divergent subsets of Amniota. Along the dashed line, the diagram traces the trajectory from the Synapsida to the crown group, Mammalia, with select representatives of various radiating groups and species, shown by the light dashed lines. Bold horizontal lines depict the appearance and duration of each taxonomic group shown. The black triangle at the Jurassic-Cretaceous boundary denotes the end of the age of dinosaurs. The geological timescale and periods appear together at the bottom of the figure which was adapted with alterations from [10].

The importance for our discussion of the proliferation of different amniote lines is that it shows that mammals did not evolve from reptiles. Rather, mammals and reptiles evolved from a common ancestry, the earliest amniotes, from which they diverged onto separate evolutionary paths more than 300 mya, denoted by the I. in Figure 1. At the demarcation of the Amniota in the figure, the divergence can be seen between Synapsida and Sauropsida. This is a noteworthy point because, as some readers will recall, early synapsids have been traditionally termed “mammal-like reptiles” ([14, 16]), a vestige of an earlier habit of calling all the early amniotes (both synapsid and sauropsid) “reptiles.” Recognizing the evolutionary distinction between synapsid and sauropsid helps to clarify that neither mammals nor mammary glands evolved from a reptile-like ancestor with a scaly, nonglandular epidermis and calcified eggshells [11].

With the clarity of retrospection, we can see the trajectory from early synapsids to mammals. Beginning in the late Pennsylvanian period, the synapsids underwent multiple evolutionary radiations and extinctions. (Figure 1); depicts some of the new forms that appeared and disappeared along the path from the early amniotes to the Synapsida and then to the Therapsida and Cynodontia. These were eras of robust speciation for the synapsids, which became the dominant forms of animal life during the Permian and Triassic periods until the dinosaurs ascended to ruling status in the late Triassic. It was about 225 mya that mammal-like synapsid forms appeared.

**Figure 2:**
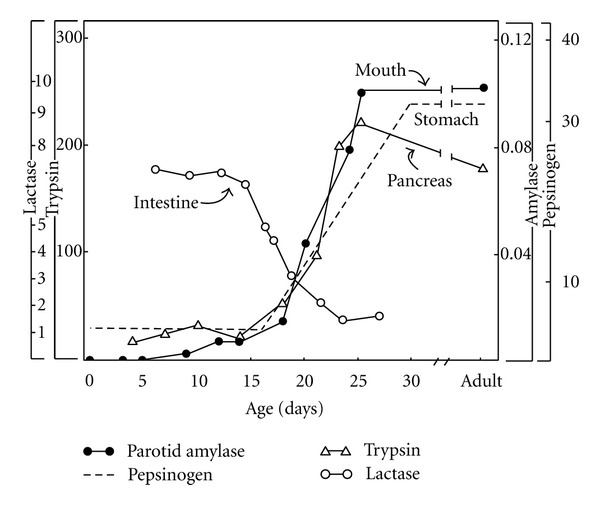
Developmental course of selected digestive elements of the mouth, stomach, pancreas, and intestine. Data were from [47, 50] and initially integrated in a discussion [51] that provides fuller reference information.

Cynodonts were among the enduring groups of synapsid (Figure 2); they became abundant during the Triassic [17, 18]. Their fossils display mammal-like traits including those specifically associated with endothermy. For example, respiratory turbinals, structures associated exclusively with conservation of respiratory water and the rapid respiratory rates needed to oxygenate endothermy, are found in the nasal cavity of cynodonts [19]. Cynodont fossils also present vascularized fibrolamellar bone, suggesting rapid rates of bone growth and remodeling. In addition, there is present in Cynodont fossils a secondary hard palate that enables nasal breathing while holding prey in the mouth—which is helpful, if not necessary for a predator. Accompanying all these features are modifications of skull, jaw, and teeth that are consistent with handling the high levels of food intake needed to support elevated metabolic rates. Together, such observations lead paleontologists to date the advent of mammals as around 200 mya. Although true mammals appear to have existed during the Jurassic and Cretaceous periods, they were not widely distributed or abundant until the Tertiary, after the catastrophic ending to the age of dinosaurs.

The evolutionary path from basal synapsids to true mammals contains many transformative changes: anatomical additions, deletions, and reshaping. All of these changes reflected and contributed to modifications in physiology and function. Among the most fundamental was the gradual evolution of endothermy, which was accomplished across the mid-Permian to the Triassic. Related to the metabolic changes associated with endogenous heat production were changes in reproductive strategy, most notably the production of smaller eggs that were retained inside the mother's body. Longer periods of egg retention are associated with the eventual evolution of viviparity [10, 14, 17, 19, 20].

### 2.2. Origins of Lactation and Oral Ingestion within the Context of Mammalian Evolution

We have rapidly recited an evolutionary trajectory from the basal synapsids to the advent of mammals. During the 200 million years between the appearance of amniotic eggs and placental mammals, lactation began and gradually became more sophisticated. Similarly, there began the oral ingestion of maternal secretions, and this behavior gradually evolved, probably in concert with the evolving maternal lactational system, to become suckling specializations that we see in contemporary mammals. In terms of origins, however, Oftedal makes the exciting and nonintuitive assertion that lactation actually predated mammals [10]! As we will see, the origin of lactation is obscured by our knowledge of its eventual role as the nutritive foundation for mammalian newborns.

We note that originally, vertebrate evolution was based on the production of independent offspring. The calcium-based structures that define the vertebrate body-spine, skeletal appendages for walking and running, thoracic rib arrangements to enable the rapid breathing needed to support sustained exercise, skulls for the evolving brain-also produced the jaws, and teeth that enabled dietary diversification. The dentition of early ancestors tells us that they were capable feeders as growing juveniles. We can see this illustrated in the basal synapsids whose teeth were replaced continuously [17, 21], suggesting that members of every age group could feed independently [17]. Indeed, such feeding independence was an imperative, for these were offspring which experienced no parental care, representative of many highly conserved and prevalent reproductive strategies that have persisted to the present.

According to Oftedal's analysis and synthesis [10, 11], mammary gland secretions first evolved in synapsids *to provide moisture to their eggs*. That is, lactation arose as a means of protecting against dessication rather than providing nutrition. Oftedal buttresses this novel hypothesis with a thorough analysis of the physiological characteristics of “parchment shell eggs,” the type most agree was produced by the synapsids and which are still produced by contemporary monotremes [11]. Oftedal's provocative suggestion would place the evolution of lactation in the Permian, some 300 mya.

Of course, the hydration of eggs through mammary secretion is not lactation as we know it today, but it is a beginning. Oftedal [10, 11] argues persuasively that lactation began well before the appearance of species we would classify as mammals. He traces the origins of lactation to reproductive strategies used in the Triassic and Jurassic, focusing on some of the mammaliaformes of that time. These were small insectivores which came in a range of sizes from about 3 g (comparable to contemporary shrews) to 25 g (mice) to 500 g (large rats). Their skeletal structures suggests that they were agile climbers (an advantage in a world dominated by dinosaurs) and probably nocturnal and elusive in their habits. These suggestions link with more specific observations supporting the idea that these early mammaliaformes were endothermic. Small endotherms require dense fur insulation and, indeed, “halos” of dense fur have been found surrounding fossils of small eutherian mammals from the early Cretaceous [22]. This is a potentially vital link to the origins of lactation, for there are a variety of proposed pathways that link endothermy to both parental behavior [20] and lactation [10].

The presence of epipubic bones in these small mammaliaformes is used as evidence for maternal care of immature offspring [10]. In addition to their role in locomotion, the sexually dimorphic epipubic bones can provide support to developing young in a marsupial pouch or to suckling young in pouchless marsupials [23]. Epipubic bones are ancient—appearing in some cynodonts and in many Mesozoic mammaliaformes [22, 24–26]. They have been lost from all extant mammals, except in monotremes and marsupials.

Oftedal projects a scenario for these small, insectivorous mammaliaformes in which the mammary patch secretions used for egg hydration became the basis of a nutrient medium for hatchlings; lactose was probably contributed by apocrine-like glands, some of which may been present in early synapsids. By the late Triassic, cynodonts were likely secreting a nutrient-rich milk, enabling a decline in egg size and an increase in altriciality of the young at hatching.

There are a host of details that support this compelling facet of evolution, but they are not vital to our discussion here. One of the central themes, however, is key: birth of immature offspring begins to develop in these early premammalian forms. Dentition is again relevant, as it was earlier when we discussed the production of independent offspring. Collectively, the fossils of these small, presumably *dependent* offspring tell us that they were diphyodonts—animals with two successive sets of teeth, the first “deciduous” set and later the “permanent” set. Most mammals are diphyodonts. The demands of diphyodonty include a period of *dependent* feeding, as well as substantial acquisition of calcium, phosphorous, and other nutrients for skeletal growth prior to independent feeding; therefore, lactation or some facultative form of nutrient provision is required. Thus, with the evolution of small, immature offspring comes the addition of a new feeding relationship and a new mode of oral ingestion.

It is noteworthy that the three taxonomic clades that comprise the existing mammals, that is, the monotremes (platypus and echidnas), metatherians (marsupials), and eutherians (placental mammals), first appear in the fossil record in the Cretaceous, but may have diverged earlier, in the Jurassic (Figure 1). It is generally accepted that oviparity (egg-laying) is ancestral to viviparity (live-bearing). It has been estimated that among reptiles, viviparity has evolved independently from a prior period of oviparity more than 100 times and never has a case of the reverse been observed [27]. Thus, it is strongly implied that monotremes use the ancestral form of parity. Evolutionarily, the progression seems to involve sequential increases in the duration of egg retention, until a condition is reached in which the egg essentially “hatches” internally, thus resulting in viviparity.

Clearly, we have drawn extensively on Oftedal's creative and scholarly efforts, for they provide a broad base of evidence for the early origins of lactation and suckling. Vorbach et al. [28] offer another perspective on the origins of lactation. They explore the idea of a development sequence in which the mammary gland evolved from simple skin glands that secreted mucuous containing a variety of antimicrobial molecules for the protection of damaged skin and only subsequently evolved nutritional components to nourish hatchlings or newborn mammals.

For our purposes, the validity of Oftedal's early hydrational origins hypothesis versus that of Vorbach et al.'s immunological protection hypothesis is not essential. In fact, the central ideas in these two innovative perspectives are not mutually exclusive and may prove to be jointly true. The essential insight, and the value of reflecting on the grand picture of mammalian evolution, is to recognize that *oral ingestion evolved at least twice*. First came independent feeding. We have considered many tens of millions of years of evolutionary history during which the many and various forms of animal life produced and reproduced offspring that hatched from eggs (fertilized externally or internally) into a world in which parental care was nonexistent and from which they had to feed independently. Thus, the sensory apparatus, motor systems, digestive capabilities, and nervous systems of all these juvenile forms were such that they were capable of immediate independent feeding. There is ample evidence of such life forms today, all descendants of ancestors common to the taxa that subsequently evolved altriciality and parental care.

Thus, brain-body systems for independent feeding is the ancestral state. Lactation and other parental secretions that can provision the young evolved subsequently. As we have seen, lactation did not evolve on its own. It was accompanied, probably facilitated by coevolutionary pressures on altriciality of offspring as well as novel adaptations in the offspring. Undoubtedly, some of the features that were or became specializations for suckling can be traced directly to adult adaptations, such as the bony secondary palate. This feature of certain synapsids, posited to have been selected as a facultative feature for predatory feeding, is an essential precondition for breathing while sucking.

Evolution, then, created and honed a constellation of specializations in increasingly altricial offspring that enabled exploitation of hydrational, nutritive, and immunological provisioning by the parents. These specializations were added to an existing system for feeding and they were added during relatively early phases of development, rather than tagged onto the end of an existing developmental sequence. In the language of heterochrony, the addition of a new feature at an early stage of development is termed “pre-displacement,” [29, 30] and this would be the case for the addition of suckling to a preexisting system for independent feeding. The result, then, is the evolution of two feeding systems—the ancestral feeding system and the specialized, limited, and more modern addition of a suckling system. In this way, oral feeding is not unitary and should be appreciated in terms of its evolution as having two separable heritages.

## 3. Suckling and Feeding as Separable Systems

To support and enrich the assertion that oral ingestion is not a unitary process, we turn to years of research, mostly from the field of developmental psychobiology. That discipline offers a broad body of well-controlled studies of the ontogeny of ingestive behavior, primarily focused on Norway rat pups. We apply that literature here, treating the rat as a decent representative of mammalian development with perhaps special applicability to the development of other generalist, omnivorous feeders-including humans. Rat development has proven value as a source of insight and understanding of human development, especially in key areas of sensory, motor, and physiological development (e.g., [31–36]). We will make the case that suckling and feeding are separable systems by systematically comparing them.  Table 2 lists the kinds of comparisons that we will review. The interested reader may consult Hall and Williams' seminal discussion in which they compare suckling and feeding in rats [37].

### 3.1. Sensory Controls

Suckling by infant rats and other nonhuman species is dependent on the sense of smell. Teicher and Blass [38] washed the nipples and ventrum of anesthetized rat mothers with a fat soluble solvent or with water. Pups presented to the ventrum of a dam washed with the fat soluble solution did not attach to any of her 12 nipples, whereas pups presented with a water-washed control dam readily attached. Impressively, when the distillate of the wash fluids was applied to the dams' nipples, nipple attachment and suckling were reinstated, thus making it clear that the critical olfactory cue(s) had been removed and replaced and eliminating explanations such as masked odors or aversive residues. The dramatic finding that nipple attachment in the infant rat is wholly dependent on olfactory cues was further supported by the effects of anosmia on pups' suckling and weight gain [39–41]. Whereas anosmia can eliminate suckling in rats, it does not disrupt feeding on solid food. In fact, pups made anosmic at a weanling age, when they will both suckle and feed independently, will fail to attach to nipple but will readily eat [39].

### 3.2. Motor Patterns

Westneat and Hall [42, p. 539] asked a basic and blunt question, “Is suckling a neuromuscular precursor to chewing, or are suckling and chewing independent systems?” They used electromyogram (EMG) recordings from superficial masseter, anterior digastric, sternohyoideus, and genioglossus muscles during suckling and chewing by rat pups at six ages, ranging from Postnatal Day (PND) 6 to 21. Suckling behavior by rats consists of nipple attachment, rhythmic sucking, and the stretch response to milk letdown. The EMG patterns were distinct for each component. Chewing EMGs were present by PND 12 and are adult-like by PND 18–21. EMG patterns during nipple attachment and adult chewing were similar, but the other elements of sucking differed from chewing. Thus, at the neuromuscular level, there are discontinuities between the sucking and feeding patterns as well as at least one EMG continuity between the forms of ingestion.

We can also look into the brain and examine the central circuits that correspond to the muscles that suck and chew, again noting the rhythmic components of both sucking and adult chewing. Here the data come from guinea pig, a species born at a relatively well-formed stage of development. The infants both suckle and, within the first postnatal week, also chew solid food. The rhythmic patterns for guinea pig oral ingestion originate from central pattern generators in the frontal cortex of the brain [43, 44]. Neurons from this cortical area project to an oral rhythm generator in medulla oblongata [45]. It was shown that there exist at least two separate pattern generators, one for sucking and one for chewing; during the transition from suckling to chewing, there is a corresponding structural and functional transition of active neurons [46].

### 3.3. Digestive Systems

 It is possible to dissociate the suckling and feeding systems in the developmental profiles of some of the digestive enzymes that comprise the postingestion physiology of the infant and weanling. The open circles in Figure 2 illustrate levels of intestinal lactase in the suckling rat, which are high throughout the first two postnatal weeks (when the rat is an obligate suckler) and then begin a precipitous decline during weaning [47] which, in this species, lasts until about PND 30 [33, 48, 49]. In contrast, the figure's triangles, broken line, and filled circles show levels of enzymes that help digest solid foods at different levels of the oro-gastrointestinal system—these levels rise together just as lactase is decreasing (cf., [50]) The coordinated changes in digestive enzymes in Figure 2 beautifully illustrate the developmental dissociation of the suckling and feeding systems [51].

### 3.4. Separable, Specific Physiological Controls of Suckling and Feeding

There are a host of endogenous hormones and peptides that modulate food intake in weanling age or older rats, but do not exert similar effects on the suckling animals. For instance, exogenous insulin is known to increase food intake and weight gain in adults, but it did not have similar effects in suckling pups [52]. Likewise, a 7-day treatment regime of ghrelin was found to increase body weight of 7-week-old rats, but the same regime applied to suckling pups did not change their weights [53]. These intriguing findings alone would be suspect because there may not have been adequate experimental measures to ensure that the maternal milk supply was sufficiently abundant to allow pups to increase their intake and accumulate additional body mass. It was also shown, however, that intraperitoneal or intraventricular administration of leptin (a putative satiety factor) decreases food intake in 28-day-old pups but does not affect suckling intake [53]. Similarly, cholecystokinin inhibits food intake in adults but does not alter suckling in 5- or 10-day-old pups [54].

### 3.5. General Physiological Controls of Suckling and Feeding Are Different

In a particularly broad and inventive study, Hall and Rosenblatt [55] equipped rat pups of various ages with an oral cannula through which they could infuse controlled amounts of milk, thus by-passing the limits of maternal milk supply. Cannulated pups attach to and suckle from the nipples of anesthetized dams, so this experimental preparation is highly versatile: the experimenter can provide measured amounts of milk directly into the infant's mouth even if the pup is attached to a nipple. A small amount of milk infused into the mouth of a pup attached to a nipple elicits the age-typical behavioral responses to a natural letdown. Pups readily ingested and reacted as though the dam had a milk letdown. Using this preparation, it was shown that infant rats PND 10 and younger do not inhibit intake when milk is delivered while pups are on-nipple. This is not an artifact of the cannulation preparation, however, because by PND 15, pups show satiety responses to the same type of milk transfer.

Friedman [56] concluded that under natural conditions with an awake, active lactating dam, it is maternal milk supply that limits the pups' intake. Satiety mechanisms in the young suckling pup are not involved. Preloads of milk or other forms of calories did not reduce milk intake by 10-day-old pups. Interestingly, Friedman [56] reported that preloading the pups with water did reduce their intake which pointed his investigation toward hydrational cues, rather than nutritive controls of early suckling. He tested two types of hydrational cues, using osmotic and volemic manipulations. Rat pups at PND 10 did not alter their suckling to an osmotic challenge, but they did increase their milk intake in response to hypovolemia induced by a subcutaneous injection of polyethylene glycol, a hyperoncotic colloid.

### 3.6. Pharmacological Dissociations of Suckling and Feeding

Our thesis is further buttressed by contrasting results on suckling and feeding obtained with pharmacological manipulations. Amphetamine is widely recognized for its anorexigenic effects. Adult rats and humans show dose-related decreases in hunger and food intake to amphetamine. In rat pups, however, amphetamine does not decrease intake [52, 57]. Suckling rat pups given amphetamine and presented with an anesthetized dam actually attached to nipples more readily and suckled more vigorously than did controls [37, 57]. Milk intake was not part of this study because the anesthetized dam did not deliver milk, but it was striking to see a drug that dramatically diminishes an adult's interest in food actually augment the appetitive elements of suckling pups.

There is an additional series of studies that provides data suggesting differential controls on suckling and feeding by the neurotransmitter serotonin (5-hydroxytryptamine, or 5-HT). Drugs that block 5-HT receptors, such as methysergide, stimulate weanling age pups to suckle. That is, they increase sucking in pups that are developmentally disinclined to attach to nipples and suck. An exciting observation in relation to our interest in dissociation of dual ingestion systems is that 5-HT antagonists cause weanling pups to prefer to suckle rather than to eat food [58]. Furthermore, the more age-typical preference for feeding can be reinstated by treatment with a 5-HT agonist [59].

### 3.7. Incidental Observations Implying Separate Central Mechanisms for Suckling and Feeding

 There are remarkably few data on the development of brain mechanisms for suckling and feeding. Historically, much attention was directed at hypothalamic control of hunger and satiety and the involvement of ascending pathways in and around the medial forebrain bundle, but such studies have largely yielded to investigations of neurotransmitters and systemic factors as noted earlier in our review. Electrolytic lesions in a variety of ingestion-related brain regions have generally had similar-appearing disruptive effects on both sucking and feeding, but such work has not been pursued developmentally in great detail.

Other forays into questions of central mechanisms for suckling versus feeding are similarly incomplete but intriguing. Bignall and Schramm [60] reported a developmental study of ingestion by “mesencephalic animals,” kittens decerebrated within one week of birth and followed up to two months of age. They reported that the abolishment of suckling behavior was the only discernible immediate deficit and that the ingestion of solid foods and lapping of milk developed at weaning age. There are many limitations to their study, including the small sample size (*n* = 5) and the fact that the surgical transection was complete in only two of the animals. Nevertheless, they reported that suckling was permanently abolished in all the animals, but by maintaining them on gavage feeding to weaning age, feeding behavior emerged on schedule. 

### 3.8. The Separate Roles of Experience in the Maintenance of Suckling and Emergence of Feeding

**Figure 3:**
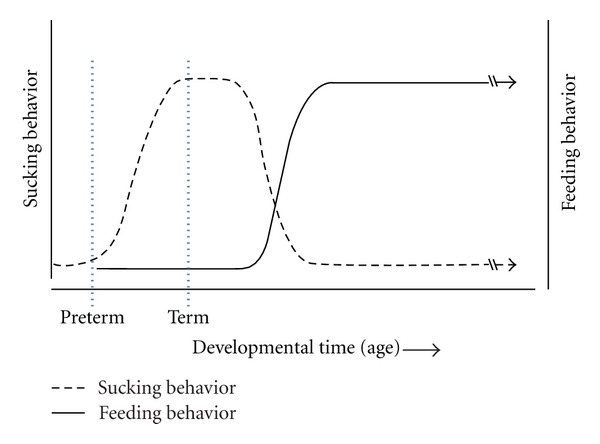
An idealized depiction of the coordinate development of suckling and feeding behavior which, together, comprise the development of oral ingestion. Note that the horizontal axis depicts an unspecified range of “developmental time” with vertical markers exemplifying a preterm birth and a term birth. The purpose of noting these events is to illustrate that the suckling system may not be functionally ready for the preterm infant, whereas it is prepared to be engaged at term.

Figure 3 is an idealized depiction of the coordinate development of suckling and feeding. The open circles depict the initial presence of suckling, its maintenance through infancy, and then its gradual decline and disappearance. The closed circles depict the gradual emergence and establishment of feeding. Typically, the two processes are synchronized (as depicted in the figure), so much so that many reasonable and thoughtful observers see the synchrony simply as the continuous development of a single system of oral ingestion. In this context, then, it was stunning when Pfister et al. [61] raised rats among litters of pups that were periodically replaced so that the subject rat was always living with suckling age pups and the mother. These subjects weaned onto solid food, but continued (even into sexual maturity) to suckle along with their preweanling counterparts! This dramatic (and bizarre) experiment tells us at least two things pertinent to the present thesis. First, the developmental dissociation of suckling dissolution from the emergence of feeding provides an important demonstration that suckling does not become feeding; the odd social context provided by these experimenters kept suckling behavior intact while the pups' feeding system separately emerged and matured. Second, it is possible to manipulate separately the ontogeny of suckling and the ontogeny of feeding, adding another type of evidence that the systems are separable.

The independence of suckling and feeding systems was echoed in a different type of experiment, the results of which strongly suggest that feeding in rats can develop without prior experience or practice of suckling behavior. Hall [62] devised a preparation whereby a cannula is installed in the stomach of a newborn rat so that it can be raised alone, typically in a container that is kept in a warm, moist environment where a milk formula is infused directly into the stomach and experimenters periodically stimulate urination and defecation. This “pup-in-a-cup” technique has been used for a variety of preliminary investigations including questions about of the role of suckling experience as a precursor to feeding. Pups raised in isolation from the mother and fed via the gastric cannula PND 18 were then tested for their ability to feed. (Cannulation was performed between 12 and 48 hrs after birth.) Remarkably, when these suckling-deprived animals were given access to solid food on PND 19, the latency to begin eating was identical to normally reared pups. Moreover, the suckling-deprived pups performed well and even equivalently to suckled pups with compensatory feeding after 24 hr food deprivation, with compensatory drinking after water deprivation, in response to dietary adulteration, and they demonstrated equivalent food motivation by learning to press a bar for food and water as rapidly as did normal rats [62].

In contrast to the feeding system's apparent independence from prior suckling experience, the suckling system appears to depend on stimulation and experience. After as little as two days of the artificial rearing regime, including the suspension of suckling experience, pups no longer display the ability or willingness to suckle [63]. In this way, suckling and feeding are further dissociated by vulnerability to disruption. The psychobiology literature contains many examples and much knowledge of how both suckling and feeding are experience-sensitive and also how the two systems interact. Here we chose to emphasize findings that elucidate the ways in which the systems are separable, for these aspects are relatively novel and can lead us to new approaches to solve old problems.

### 3.9. Learning Mechanisms That Separate Suckling and Feeding

One of the most rapid and enduring associations that rat pups can learn are “conditioned taste aversions.” Such learning occurs when a distinct and novel taste is followed by a treatment that causes nausea or gastrointestinal malaise, typically induced by a dose of a nonlethal toxin such as lithium chloride (LiCl). In one part of a series of experiments [51, 64, 65], rat pups were equipped with the type of oral cannula described earlier [55] that enabled precise amounts of flavored milk to be delivered by an experimenter. When milk containing a novel flavor was pulsed into a pup's mouth and this flavor experience was paired with LiCl-induced illness, the pup would subsequently avoid ingesting a food (powdered rat chow) containing the illness-associated flavor. The association was learned in one trial and the learning was strong and enduring. Remarkably, if the pup was attached to a dam's nipple when the same flavored milk was delivered to the same site of the tongue in the same amount, on the same schedule, and paired with LiCl-induced illness, the pup did not react in any way that indicated its memory of a taste aversion. A variety of control studies established that the key variable was whether the pup was in a suckling context. Like the painful shock study described earlier, the suckling context redefines the pup's learning contingencies and negative associations are not formed. This type of surprising finding has been discussed in a variety of ways [51, 66, 67] but for present purposes, it is a view of a learning mechanism that is functionally defined by whether it occurs in the context of suckling or of feeding.

### 3.10. Independent Ingestion by Neonatal Rat

A neonatal rat pup, at PND 3, deprived of nutrition for nearly a day and placed alone in a warm, humid chamber with a puddle of milk or wet mash on the floor will, under such conditions, orally ingest milk from the puddle. It is argued that this remarkable phenomenon is a case of regulated, independent ingestion because the behavior ceases when the pup ingests about 5% of its body weight; impressively, the amount the pup ingests is diminished by a preload of milk [68]. Recall that rat pups 10 days old and younger are demonstrably nonregulatory when they are tested on nipple. A popular interpretation of independent ingestion by an isolated 3-day-old infant rat is that it demonstrates that there exists a system for independent feeding in the neonatal brain that is normally inaccessible, further evidence of a dual ingestive system in a developing mammal.

## 4. Do Human Infants Possess Dual Ingestion Systems?

Treating oral ingestion as an aspect of behavioral biology makes it obligatory to consider its the evolutionary and developmental aspects. Within a biological framework, we expect the ontogeny of oral ingestion in humans to be consistent with that of other mammalian species, and that human infants also possess “dual ingestive systems.”

The dual ingestive system thesis is novel, particularly in clinical domains, so it is understandable that there are few data such as those known for rodent development. Nevertheless, it is possible to note several lines of evidence that are consistent with our assertion that dual ingestive systems are a conserved feature of mammalian development that pertains to all mammalian species, including humans. Human infants, like the offspring of every mammal, begin postnatal life as monophagous organisms (those that ingest only one substance), deriving all nutrition, water, and electrolytes from a single source—mother's milk. Suckling is the singular mode of ingestion while in the developmental phase of monophagy. As in other newborn mammals, suckling is the obligatory, initial phase of ingestion for humans.

Feeding, the ingestion of nonmilk foods including solids and boluses of soft substances, typically has a gradual onset after suckling is established. In human offspring, as in most mammalian young, suckling continues to be displayed even after the onset of feeding. In fact, the developmental duration of overlap of the two modes of ingestion can be considerable, depending on a variety of contextual variables, including food availability and cultural traditions. Thus, human infants may suckle exclusively for six months and then both suckle and feed for another six months or more. Different substances from different sources are taken with each mode of ingestion. Human suckling and human feeding behaviors are distinguishable in terms of their topographies. A host of morphological changes and muscular developments underlie age-related modifications of the tongue, and oral-pharyngeal complex helps differentiate the movements that comprise sucking and chewing in human infants (see [69] for a useful review). Similarly, the neural systems that mediate sucking (and coordinate breathing and swallowing) are separable from many of those that mediate feeding (see [69]). In such ways, as we have seen in analyses of nonhuman ingestive development, suckling does not transform into feeding; both modes of ingestion are expressed separately within the same developmental phases. That the absolute duration during which the two behaviors exist in an infant can vary greatly also suggests that feeding develops independently of suckling in humans.

Understandably, experimental studies involving some of the more dramatic manipulations imposed on laboratory species are not conducted with humans. Importantly, however, Pickler et al. [2] provide nonexperimental evidence that achieving competence with nipple feeding depends on the amount and timing of prior experience. Such observations connect importantly to our general knowledge that suckling is a complete, adaptive, integrated behavior with its own functional integrity that is shaped by experience and context.

Although we emphasize that suckling and feeding are separable along many dimensions, the two behaviors are *not separate*. The prevalence of feeding disorders among children who sustained disrupted suckling experiences [70] is an important reminder that we must pursue deeper understanding of the connections between suckling and feeding. Menella and Beauchamp's studies (e.g., [71–73]) showing that flavor experiences during suckling or even in utero can affect feeding preferences in later life indicate that there are experiential lines of continuity between the two ingestive systems.

## 5. How Does the Thesis of Dual Ingestion Systems Relate to the Development of Oral Feeding in the NICU?

Lactation and suckling are unique to mammals and universal among them, thus squarely placing this aspect of human reproduction and development in a broad biological context and directing our attention to evolutionary history, for this is the source of insights into comparative questions. When we examine the evolution of lactation and oral ingestion we discover beautiful continuities, as we should with any feature shared by diverse species with common ancestors.

The evolutionary story tells us that infant suckling is a “new invention” that followed the advent of live birth and immature offspring. Our evolutionary ancestry is from externally delivered eggs which hatched relatively well-formed offspring capable of independent ingestion. We saw that suckling was subsequently “invented” and therefore added to the pre-existing systems for independent, oral ingestion. When we examined the development of oral ingestion in a living relative of ours—a laboratory rodent—we discovered a swath of evidence for two ingestive systems, one for sucking and one for feeding [37]. They are separable, though the two ingestive systems are not entirely separate.

The perspective on the evolution and development of oral ingestion, as outlined here, has value and validity for practitioners and researchers concerned with oral ingestion in human infants. The perspective we have introduced can increase and sharpen our awareness of the developmental structure of oral ingestion [1–3, 7]. This alone can help define areas where knowledge is needed and help prioritize what knowledge we should apply. As such, we are better able to treat suckling as a complete behavior, not as practice for a subsequent behavior or as a precursor to feeding, but as a complex, ontogenetically complete behavior with an adaptive integrity to be attained and then followed by developmental dissolution. With the adoption of such a view, a new set of research questions can be articulated and take on a new importance. When suckling is seen as a developmental goal, the *precursors of suckling* become germane, especially to clinicians who work with premature infants whose suckling is undeveloped.

For example, swallowing might now be viewed as an important behavioral precursor to the onset and development of suckling. We know that fetuses swallow and that the behavior serves a variety of immediate and prospective functions [74]. How often, when, and how much do fetuses swallow? Does the premie swallow similarly? It seems likely that an infant, prematurely thrust into an environment where nutrients are delivered to its stomach via nasogastric tube and pulmonary breathing is not only required but is assisted with forms of pressurized airflow, will adopt patterns of swallowing different than that of a same-aged fetus. We would probably serve the infant well to understand this in greater detail.

Within the new framework, chemical cues (tastes and smells) that contribute to state, activity, orientation, arousal, and calming [75–77] and that can initiate digestive processes now have magnified potential. Through the lens of the “dual ingestive systems,” we can see with greater clarity their possible importance. We are compelled to ask if there is important information in knowledge of the tastes and odors of amniotic fluid and vernix and glandular secretions of mothers and fathers. Can we use such odors to augment state regulation in the infant or to stimulate ingestion? Can we better see the natural contingencies for learning when a mother brings her baby to the breast, stimulating its senses and coaxing from it a series of new acts that are rewarded with external and internal sensations? In the context of suckling as a complete developmental system, it is possible to envision how odors might be used in the NICU to recreate some of the key contingencies and associations and facilitate the preterm infant's developmental course.

The baby's experiences are particularly significant in this framework. Experience and action are vital components in modern developmental analyses [78–81]. Our thesis provides a framework that can help order and organize experience into elements that can separately contribute to suckling and later to feeding. One kind of experience may have similar or different effects on either or both of the two kinds of ingestion. The experimental literature can show how to recognize and to differentiate among such kinds of effects.

These are but a small sample of some of the practical implications and promises for improved practice that could evolve from the thesis we have introduced in this paper. There is need for further research, so that we might better help infants develop suckling as part of an integrated array of adaptive competencies that will enable discharge from NICU to home [3, 4, 6, 7]. As with any new formulation, it is certain that important qualifications and unforeseen implications await us. But only by adopting and applying these ideas and pursuing with research their value and utility will we be able to make the most of them and understand the extent to which and how clinical practice and, most importantly, the outcomes on babies and their families, can be improved.

## Figures and Tables

**Table 1:** Table of geological timescales used in this paper. The nomenclature and dates are based on those in [12, 13].

Cenozoic era	
Quarternary period	1.6–0 Myr
Tertiary period	66.4–1.6 Myr
Mesozoic era	
Cretaceous period	144–66.4 Myr
Jurassic period	208–144 Myr
Triassic period	245–208 Myr
Paleozoic era	
Permian period	286–245 Myr
Carboniferous period	360–286 Myr
Pennsylvanian (later)	
Mississipian (earlier)	
Devonian period	408–360 Myr
Silurian period	438–408 Myr
Ordovician period	505–438 Myr
Cambrian period	570–505 Myr

**Table 2:** Comparisons applied to sucking and feeding behaviors that are used to evaluate whether the development of oral ingestion is better understood as a unitary process or should be dissociated into separate developments.

Sensory controls	
Motor patterns	
Digestive systems	
Hormones and peptides cues	
Physiological regulations	
Pharmacological manipulations	
Brain mechanisms	
Experiential mechanisms	
Developmental timing	
Specializations of learning	
